# Analyzing Visual Metaphor and Metonymy to Understand Creativity in Fashion

**DOI:** 10.3389/fpsyg.2018.02527

**Published:** 2019-01-07

**Authors:** Ryoko Uno, Eiko Matsuda, Bipin Indurkhya

**Affiliations:** ^1^Division of Language and Culture Studies, Institute of Engineering, Tokyo University of Agriculture and Technology, Tokyo, Japan; ^2^Division of Advanced Information Technology and Computer Science, Institute of Engineering, Tokyo University of Agriculture and Technology, Tokyo, Japan; ^3^Department of Cognitive Science, Institute of Philosophy, Jagiellonian University, Kraków, Poland

**Keywords:** fashion design, creativity, cognitive linguistics, metaphor, metonymy

## Abstract

The role of figurative language such as metaphor and metonymy in creativity has been studied in cognitive linguistics. These methods can also be applied to analyze non-linguistic data such as pictures and gestures. In this paper, we analyze fashion design by focusing on visual metaphor and metonymy. The nature of creativity in fashion design has not been fully studied from a cognitive perspective compared to other related fields such as art. We especially focus on fashion design as a communication tool between the designer and audience in conveying a designer’s image of human beings. Photos from two fashion shows were analyzed. We carried out an experiment to compare how human images in two shows are interpreted by those who are familiar with fashion and those who are not. We obtained three results: (1) As far as figurative (metaphorical and metonymic) interpretations of human images are concerned, two groups with different levels of familiarity with fashion had significantly different patterns of responses to two shows. (2) For the non-figurative interpretations (such as physical or personal attributes), no significant difference in the pattern of response to the show was observed between the two groups. However, the participants as a whole responded to the two shows differently. (3) In addition, for the non-figurative interpretations, the fashion experts found significantly more attributes in human images than the other group. The results show that the analysis of figurative interpretations is effective in understanding how familiarity with fashion affects the mode of seeing fashion shows.

## Introduction

An influential designer in the fashion industry today, Karl Lagerfeld, once said “Fashion is a language that creates itself in clothes to interpret reality.” Fashion design is an act of creating what people put on, and people are always presupposed in this process. Thus, to design fashion is to design the human image that sustains this fashion. Lagerfeld’s declaration that fashion interprets reality also suggests that fashion proposes an image of the human whose fashion reflects reality. When we focus on fashion as a creation of human images, we make a case to examine fashion within cognitive science.

Following Lakoff and Johnson (1980), the role of figurative language, especially metaphor, in creative thought has been widely studied. Not only verbal but also visual metaphor and metonymy have been analyzed to reveal dynamism in conceptual structures (Indurkhya and Ojha, 2013). This paper seeks to reveal creativity in fashion by focusing on visual metaphor and metonymy. Compared to related fields such as art or product design, creativity in fashion has not received much scholarly attention. For example, in the *Cambridge Handbook of Creativity* (Kaufman and Sternberg, 2010), while there are independent chapters on art (Locher, 2010) and product design (Cropley and Cropley, 2010), fashion does not even appear in the index.

Not many studies of fashion creation take on a cognitive perspective, although some have employed a sociological perspective. Kawamura (2005) proposed a field of study called “fashion-ology,” pointing out that fashion and clothing are two different things: the former, which is invisible, is realized through the latter, which is visible. The origin of the term “fashion,” as it is currently used, can be traced to 1489 (Brenninkmeyer, 1963). Though fashion was limited to the upper class gentry in the 15th century, it became more democratic in the 20th century. This is only one of the many aspects telling us that the meaning of fashion has shifted over years. Nonetheless, “change” and “novelty” are still regarded as two main characteristics of fashion (Kawamura, 2005).

Even in the sociological context, there is no consensus on whether fashion can be classified as art (Miller, 2007). Moreover, Skov et al. (2009) argue that even when we see fashion as art, there are two different interpretations. On one hand, fashion can be seen as an everyday art form. For example, Wilson (1985) describes fashion as a way to express modernity, even by ordinary people. On the other hand, we can see fashion designers as artists. In this perspective, the most important event in fashion is the fashion show: it is an event where the fashion designer is in complete control of esthetic concepts. Fashion shows began by conveying the image of fashion to end customers. However, their role has changed since the 1960s, with the rise of fashion photos and magazines (Moeran, 2006), and the press became the main audience of these shows, freeing them from having to promote sales and making them autonomous (Skov et al., 2009).

In this paper, we take a cognitive scientist’s point of view to capture the essence of fashion, i.e., the creation of human images, and follow the abovementioned position that regards the designer as an artist. We use photos from fashion shows, for fashion shows reflect the designer’s images directly, as argued by Skov et al. (2009). We understand the show as a setting to communicate human images from the designer to the audience through the design medium. In this case, the designer and the trained audience share creativity in fashion. In the research presented here, by focusing on visual metaphor and metonymy, we examine how photos from fashion shows are variously interpreted by those who are familiar with fashion compared to those who are not.

### Visual Metaphor, Visual Metonymy

In cognitive linguistics, the role of figurative language, especially metaphor, in creativity has been studied (Lakoff and Johnson, 1980; Indurkhya, 1992). Metaphor is a non-literal expression that uses a form to express a meaning that is similar to the literal meaning, an example being “time is money.” Literally, time is not money. The reason why time is said to be money in this expression is because there is a common property between them: being “valuable.” Thus, to understand the concept of time, we use the concept of money. It is a mechanism to expand our understanding of things by utilizing already acquired knowledge. Lakoff and Johnson (1980) point out that mapping the knowledge structure of money to that of time is happening at the conceptual level, not the verbal level. To confirm this point, Thibodeau and Boroditsky (2011, 2013) carried out experiments to show that by presenting different metaphors, we can affect people’s choice of actions. As Forceville (2009, 2016) notes, if metaphor is conceptual, it cannot be limited to language. Among cases of non-verbal metaphor, the most studied are those in pictures and gestures. Visual metaphor has been studied in comics, advertisements, and films but not in fashion. For example, in Japanese comics, background drawings can metaphorically represent the mental state of the protagonist (Shinohara and Matsunaka, 2009). Consider an example of visual metaphor in advertisement from Indurkhya and Ojha (2017). In the image shown in Indurkhya and Ojha (2017), 29, the man at the center is wearing headphones and is listening to music at an airport. He is surrounded by people whose heads are replaced with loud speakers. Here, the visual metaphor is formed by mapping loud speakers to human heads. This suggests that the sound emitted by people is as loud as the sound from a loud speaker. In other words, people are noisy. The property of a loud speaker (“emitting loud noise”) can be naturally projected onto a human being (“noisy person”).

In figurative language, metaphor and metonymy are the most widely studied phenomena. Metonymy is also a non-literal expression, but here, the shift from the literal meaning to a figurative meaning is based on contiguity, not similarity. For example, when we say, “We need more hands,” we mean that we need more people who can help us, not only their hands. We can refer to humans as a whole by their parts such as hands or heads because they are close. Another technique of metonymy is referring to a person by what he or she is wearing. The famous “little red riding hood” is a good example. It refers to the girl, not her hood. What we put on is close to us. While metaphor functions by expanding our understanding, metonymy functions via landmarks (Langacker, 2008; Nishimura, 2008). The metonymy can also be observed in other modalities: Catalano and Waugh (2013) analyzed both verbal and visual metonymies and showed how they contributed to shaping public opinion.

In brief, both metaphor and metonymy are seen in visual modality and contribute to changing the cognitive state of people. However, they have not been applied to analyze human images in fashion shows. In the present research, our goal is to investigate how an expert fashion show viewer understands the fashion show differently from a non-expert viewer by focusing on figurative (i.e., metaphorical and metonymic) and non-figurative interpretations. Our results show that it is only by paying attention to the figurative interpretations that we can understand the characteristics of the way people who are familiar with fashion see human images in fashion shows.

## Materials and Methods

### Ethics Statement

This study was approved by the Institutional Review Board (IRB) of Tokyo University of Agriculture and Technology, Tokyo, Japan. All participants gave written informed consent in accordance with the Declaration of Helsinki.

### Participants

Ninety-six participants took part in the experiment. This included 24 students from a private fashion design school (62.5% female) and 72 university students majoring in scientific fields (47.22% female). Henceforth, we call the former set of students the *fashion group* and the latter the *non-fashion* group. The fashion group participants were between 16 and 30 years old (average 25.65 years), and the non-fashion group participants were between 19 and 22 years old (average 19.68 years). The difference in the age groups between the two sets of participants reflects the differences between their schools. In Japan, most students enter undergraduate school just after finishing high school, which is at the age of 18. Conversely, in fashion schools, students come from various backgrounds, and many of them have been in employment. The participants were all Japanese except for two Korean students in the fashion group and one Chinese student in the non-fashion group. We did not check the personal cultural backgrounds of the participants in the experiment, but the Japanese students in both schools were mostly brought up in Japan. All participants were proficient in Japanese, which ensured that they understood the instructions. They participated in the experiment in their own classrooms.

### Stimuli

**FIGURE 1:**
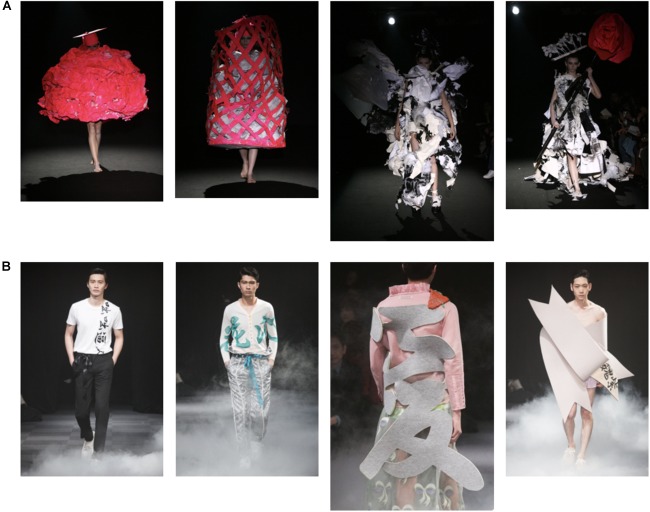
Photos presented in the experiment: Photos from the two fashion shows: the garbage show **(A)** the letter show **(B)**. The larger photos are the target photos. These images are being reproduced with the permission of the copyright holder [**(A)** writtenafterwards inc; **(B)** Fashionsnap.com]. The original sources appeared in **(A)** http://www.writtenafterwards.com; **(B)** https://www.fashionsnap.com/.

Two sets of pictures, each from different fashion shows, were chosen and shown on the question sheet (permission was obtained from the copyright holders). In each picture, a model walking the runway was shown from his or her front or back. One show was the 2009–2010 Autumn Winter Collection by *writtenafterwards* with the designer Yoshikazu Yamagata. Figure 1A shows the photos chosen from this show. The other show was the 2014–2015 Autumn Winter Collection by *Mikio Sakabe* with designers Mikio Sakabe and Shueh Jen-Fang. Figure 1B shows the photos used in the questionnaire from this show. Both shows were held during the Japan Fashion Week in Tokyo. All three designers teach at the private fashion school where the fashion group participants studied.

Though the designers featured in the two sets of pictures mainly sell their products in Japan, they are internationally recognized. For example, Yamagata and Sakabe were shortlisted for the LVMH Prize in 2015 and 2016, respectively. The LVMH prize is launched by LVMH Moët Hennessy Louis Vuitton SE in order to honor young fashion designers around the world. In addition, all three designers were educated in Europe. Yamagata studied at Central Saint Martins College of Arts and Design (current Central Saint Martins, University of the Arts London) in the United Kingdom, Sakabe at the Royal Academy of Fine Arts, Antwerp, Belgium, and Shueh at La Cambre in Brussels, Belgium. Yamagata and Sakabe both graduated at the top of their respective classes.

Each set contained a target photo: the participants were asked to describe the human image in the photo. A further three photos in the set were presented to the participants so as to contextualize the target photo. We used three criteria to choose the target pictures. First, the show must target displays of the human image and not just clothes. Second, since the current research analyzes the distribution of metaphorical and metonymic interpretations, the picture must be open to both kinds of interpretations. Third, two target photos must be distinctly different so as to determine whether the participants responded differently to them.

We chose two pictures that met these criteria as target pictures. To begin with, Yamagata and Sakabe (2013), who were the designers of the target photos, explicitly mentioned in a book they co-authored that they present human images at fashion shows: on the cover of this book is written, “Beyond making clothes, we make a new human.” Moreover, these pictures can be interpreted both metaphorically and metonymically. Metonymic interpretations of clothes are not difficult because the closeness between the clothes and the person wearing them is apparent. However, not many fashion photos can easily induce metaphorical interpretation. In the first target photo (the large photo in Figure 1A), instead of ordinary clothes, the model is wearing some garbage that was originally collected from architecture departments at universities. We can say that the garbage replaces the clothes, just like speakers replace heads as in the example presented before. In the second target photo (the large photo in Figure 1B), we can say that instead of clothes, the model is wearing a letter on his back. This is a Chinese letter used in Japanese and means “love.” Finally, the two shows are distinctive. When we compare garbage and a letter, the former is a substance (or a texture), and the latter is an object. In addition, the former is concrete, and the latter is abstract.

Henceforth, we call the former show the *garbage show* and the latter show the *letter show*. Among the participants, only one from the non-fashion group knew of the garbage show before the experiment. No one knew the letter show. Conversely, among the fashion group, twelve participants knew about both shows, four knew only the garbage show, and one knew only the letter show.

### Procedure

The participants were asked to answer two questions on the questionnaire by writing freely. Each side of the paper had four photos from one of the two collections, as shown in Figure 1. The photo images were all in color. Two types of sheets were prepared: the first type presented the garbage show first, while the other presented the letter show first. We randomly assigned either one of the sheets to the participants. For each set of pictures, we asked: “The following four pictures are from a fashion show. Look at the third large photo and answer what kind of *human* is represented by this photo.” We had additional information on the questionnaire for the participants. For the garbage show photos, there was an explanation that the material used for the clothes was garbage. For the letter show photos, there was an explanation that read that what was on the back of the model was a letter.

## Results

### Ratings for Figurative and Non-figurative Interpretations

After all the data were collected, the answers (freely written descriptions of human images represented by the two target photos) were rated in six categories by three coders who worked separately. There were two groups and six types of categories to rate. The first group included categories that showed how the figurative interpretations of the human images were working. The figurative group included the categories “metonymic” and “metaphorical.” These were the two main categories observed in this research. To determine how figurative interpretations work, we also put together the non-figurative interpretations group. This second group comprised four categories characterizing human personality: physical, personal, relational, and social role. This categorization was based on Smilek et al. (2007), who analyzed the characteristics of a personality description of an object by a participant with synesthesia. The details of the ratings by each coder are shown in the Supplementary Material.

Among the answers by the participants, those that did not describe a human image at all were excluded: for example, answers such as “I don’t know” or comments about the designer. For the remaining answers, the coders were asked to rate how well each answer (the description of human image) matched the attributes in the six categories. The rating was carried out using a six-point Likert scale (1, *matches completely*; 6, *does not match at all*).

Each category was explained to the coders via an instruction sheet. The descriptions of the six categories provided to the coders are given below. Some examples from the participants’ answers are also shown below after the descriptions of each category. These eight examples were all recognized by the coders to generally match their respective categories. Two coders gave a 1, and one coder gave a 2 for the answers in (2, 3, and 4). The coders rated all the remaining examples as 1.

For the transcription of the Japanese examples, the Hepburn system of Romanization was used. The following abbreviations were used in the word-to-word translations: ACC for accusative, COMP for complementizer, GEN for genitive, NOM for nominative, PASS for passive, PAST for past tense, and POL for polite.

For the metonymic category, the instruction sheet for the coders instructed that the answer matches this category when it expresses a person who is close (or contiguous) to garbage or a letter. It is also noted that this “closeness” is physical, such as spatiotemporal proximity or causation. Thus, closeness does not include similarity. Another noteworthy point is that for the letter collection, not only should we consider the closeness to the letter but also the closeness to the concept of “love” (which is the meaning of the letter). This category includes someone who has garbage or the letter (or the concept of love) on his or her body or someone who is staying close to garbage or the letter (or the concept of love), etc. Example (1) expresses a person who is close to garbage and (2) expresses a person who is close to the letter.

(1) Metonymic
  tsukaeru mono wa hirot-teshimau youna hito to kanji-masu
  useful thing TOP pick.up-PERF like person COMP feel-POL
  “I feel that she is a kind of person who picks up whatever she finds useful.”
(2) Metonymic
  mukashi no bushoh no kabuto no ai no
  old.days GEN warrior GEN helmet GEN love GEN
  imeeji ga tsuyoku isamashii hito ni kanji-ta
  image NOM strong gallant person as feel-PAST
  “The letter ‘*ai* ( = love)’ strongly reminds me of the letter on the warrior’s helmet in old days, so I felt him as a gallant person.”

The second example requires some explanation. In old Japan, military commanders wore a helmet with a Chinese character on the front as a part of the design. One of the famous commanders wore a helmet with the letter *ai.* The answer in (2) refers to this helmet.

Next, for the metaphorical category, the coders were instructed that the answer would match the attribute in the metaphorical category when it expressed a person who is similar to garbage or the letter (or the concept of love). The answer in (3) is an example that was judged by a coder to describe a person as being similar to garbage, i.e., someone who can be abandoned by society.

(3) Metaphorical
  shakai no haguruma to-shite tsukai sute-rareru hito
  society GEN cog as use discard-PASS person
  “A person who is used and discarded as a cog in a wheel in society.”

The following example (4) was judged by a coder to describe a person who is similar to the letter:

(4) Metaphorical
  shuchoh no tsuyoi hito
  assersion GEN strong person
  “An assertive person.”

For the non-figurative group, we followed the categorization used in the study by Smilek et al. (2007), which analyzed the personification of inanimate objects by a participant with synesthesia. The instruction sheet provided some examples of each category to the coders as follows: Physical attributes include features such as male, in 40s, black hair, Japanese, etc. Personal attributes include serious, optimistic, mischievous, etc. Relational attributes include friendly, dominant, etc. Finally, social role attributes include younger brother, god, leader, etc. The following are some examples judged by the coders as belonging to each category:

(5) Physical
  kireina onna-no-hito
  beautiful woman
  “A beautiful woman.”
(6) Personal
  kurai hito
  dark person
  “A gloomy person.”
(7) Relational
  mawari to no kyohchoh yori-mo
  people.around with GEN cooperation rather.than
  jishin no ishi o sonchoh-suru kanji ga shi-masu.
  oneself GEN will ACC respect feeling NOM do-POL
  “I feel that this person respects her will more than cooperating with people around her.”
(8) Social role
  yakuza
  gangster
  “A gangster.”

After all the answers were rated by the coders, two procedures were followed before the analysis. First, we converted six-point ratings into binary values. Ratings 1–3, which were affirmative to the matching, were replaced with the value 1. Ratings 4–6, which were negative evaluations of the matching, were replaced with the value 0. Next, we merged the three ratings (one by each coder) for each answer as follows. When there was a 1 by any coder in a given category, the final rating was set to 1. Only when the ratings by all the three coders were 0 was the final rating set to 0.

### Analysis of Figurative Interpretations

In this part, we focus on the figurative interpretations, namely, metonymic and metaphorical categories. As noted above, the answers to each question were converted into binary values for each of the two categories (metonymic or metaphorical). We refer to these binary values as “category degrees” for further analysis.

Taking the category degree for the objective variable, a three-way ANOVA was performed for the photos (garbage or letter), participant groups (fashion or non-fashion), and category types (metonymic or metaphorical). There were significant interactions among the photos, the participant groups, and the category types [α = 5%, *F*(1, 370) = 9.04, *p* = 0.0028], and between the photos and the category types [*F*(1, 370) = 4.11, *p* = 0.043]. No significant interactions were observed between the photos and the participant groups [*F*(1, 370) = 1.35, *p* = 0.25] or between the participant groups and the category types [*F*(1, 370) = 2.40, *p* = 0.12]. Therefore, we analyzed the data from the fashion and non-fashion participant groups separately so as to find the sub-effects between the photos and category types. For each participant group, a two-way ANOVA was conducted for the photos and category types.

**FIGURE 2:**
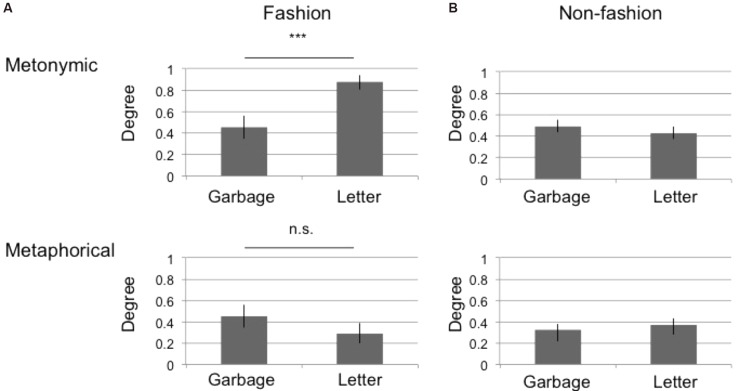
Mean tag degree across all participants. The *x-*axis denotes the photo (garbage or letter), while the *y-*axis indicates the mean category degree. The error bars indicate SEM. The results of two-way ANOVA are summarized by the notation ^∗∗∗^*p* < 0.01, n.s. *p* ≥ 0.05. **(A)** Results from the fashion group. **(B)** Results from the non-fashion group.

Regarding the fashion group, there was a significant interaction between the photos and the category types [α = 5%, *F*(1,88) = 9.2933, *p* = 0.0030]. We therefore proceeded to analyze the simple main effects of the interaction. Figure 2 shows the mean category degree for metonymic and metaphorical categories, respectively (Figure 2 upper: metonymic, lower: metaphorical). The *x-*axis denotes photos (garbage or letter), and the *y-*axis indicates the mean category degree. The error bars denote SEM. The results of the simple main effects show a significant difference for the metonymic category [*F*(1, 88) = 9.6561, *p* = 0.0025] but not for the metaphorical category [*F*(1, 88) = 1.4491, *p* = 0.2319], which are summarized in Figure 2 by the notation, ^∗∗∗^*p* < 0.01, n.s. *p* ≥ 0.05.

Regarding the non-fashion group, the mean category degree for each statistical group is depicted in Figure 1B. There was neither any significant interaction between the photos and category types [α = 5%, *F*(1, 282) = 0.9567, *p* = 0.3289] nor main effects in the photos [*F*(1, 282) = 0.0096, *p* = 0.9222] and category types [*F*(1, 282) = 3.748, *p* = 0.0539].

### Analysis of Non-figurative Interpretations

In this section, we focus on the remaining categories reflected in the non-figurative interpretations. The categories were physical, personal, relational, and social role. Similar to the figurative interpretations, we used the category degree for our analysis.

Taking the category degree for the objective variable, a three-way ANOVA was performed on the photos (garbage or letter), participant groups (fashion or non-fashion), and category types (physical, personal, relational, and social role). There were no significant interactions among the photos, the participant groups, and the category types [α = 5%, *F*(3, 740) = 0.72, *p* = 0.54], between the photos and the participant groups [*F*(1, 740) = 0.39, *p* = 0.53], or between the participant groups and the category types [α = 5%, *F*(3, 740) = 1.80, *p* = 0.15]. A significant interaction was observed between the photos and the category groups [*F*(3, 740) = 8.03, *p* < 0.0001]. Significant main effects were observed in the photos [*F*(1, 740) = 6.4678, *p* = 0.011], the participant groups [*F*(1, 740) = 16.86, p < 0.0001], and the category types [*F*(3, 740) = 81.58, *p* < 0.0001].

**FIGURE 3:**
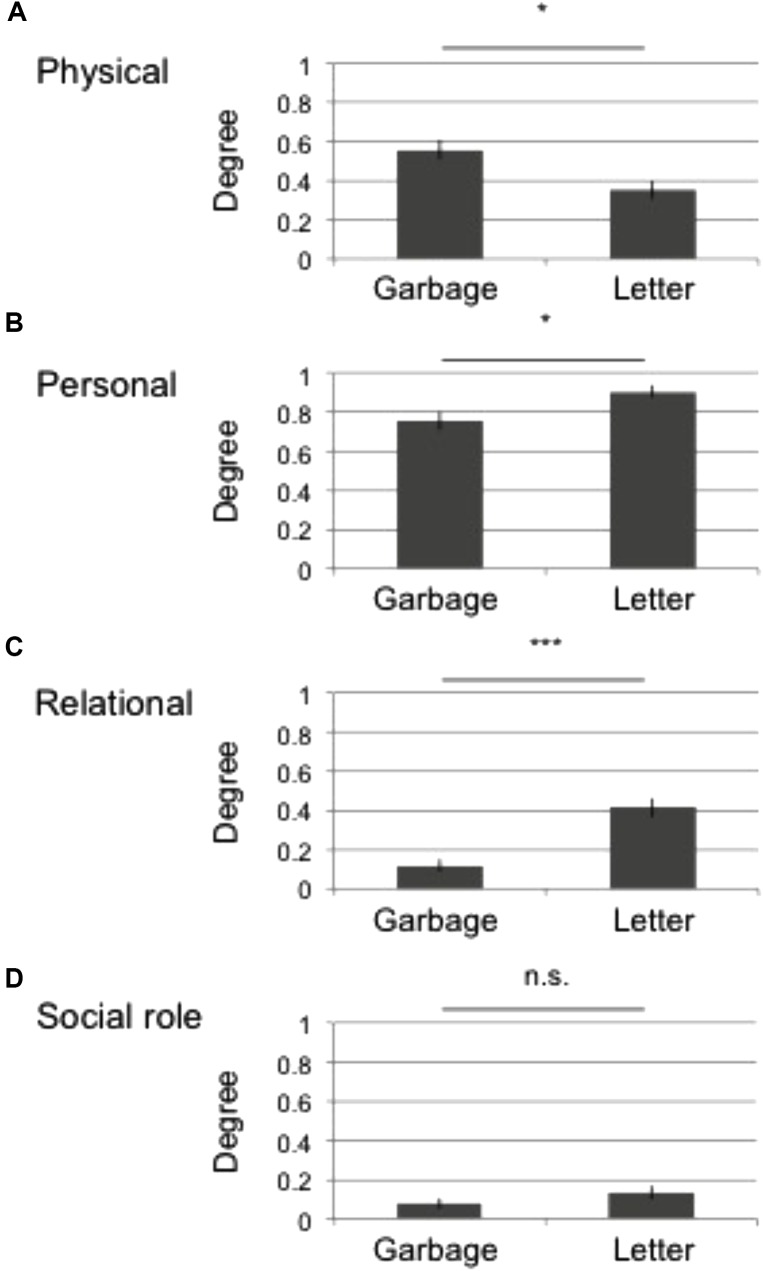
Mean tag degree across all participants. The *x-*axis denotes the photo (garbage or letter), while the *y-*axis indicates the mean category degree. The error bars indicate SEM. Each sub-figure shows the results for each category: **(A)**, physical; **(B)**, personal; **(C)**, relational; and **(D)**, social role. The results of two-way ANOVA are summarized by the notation ^∗∗∗^*p* < 0.01, ^∗^*p* < 0.05, n.s. *p* ≥ 0.05.

We therefore focused on the simple interaction between the photos and the category degrees without taking the participant groups into account. Figure 3 depicts the mean category degree for each category (a: physical, b: personal, c: relational, d: social role). The *x-*axis denotes photos (garbage or letter), and the *y-*axis indicates the mean category degree. The error bars denote SEM. The results of the simple main effects show a significant difference for the physical category [α = 5%, *F*(1,740) = 5.4153, *p* = 0.0202], the personal category [*F*(1,740) = 3.9691, *p* = 0.0467], and the relational category [*F*(1, 740) = 20.3284, *p* < 0.0001], but not for the social role category [*F*(1, 740) = 0.8327, *p* = 0.3618]. The results are summarized in Figure 3 by the notation, ^∗∗∗^*p* < 0.01, ^∗^*p* < 0.05, n.s. *p* ≥ 0.05.

**FIGURE 4:**
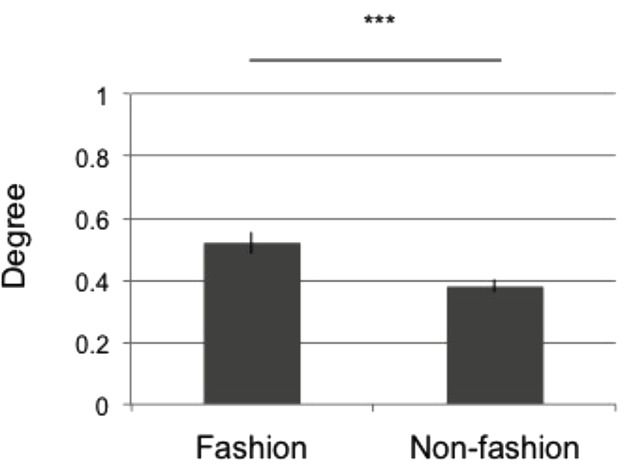
Mean tag degree across all participants. The *x-*axis denotes the participant group (fashion or non-fashion), while the *y-*axis indicates the mean tag degree. The error bars indicate SEM. The results of two-way ANOVA are summarized by the notation ^∗∗∗^*p* < 0.01, n.s. *p* ≥ 0.05.

We further analyzed the main effects of the participant groups. Figure 4 depicts the difference in category degree across the participant groups.

## Discussion

While creativity in art and design has been studied in cognitive science, fashion design has received little attention. In this study, by applying studies in visual metaphor and metonymy, we investigated the creativity of people familiar with fashion design in watching fashion shows.

We analyzed the interpretations of the human images represented in photos from fashion shows presented to two groups: people with and without knowledge of fashion. When we focused on figurative (metaphorical and metonymic) interpretations of these photos, we found some key differences in how the two groups responded to the two shows. The fashion group had more metonymic interpretations for the letter show, but there was no significant difference between the metaphoric and metonymic interpretations for the non-fashion group. Regarding the non-figurative interpretations, no significant difference was found between the two groups in interpreting the two shows. Overall, the participants found more outer attributes in the garbage show photos and more inner attributes (personal and relational) in the letter show photos. In addition, the fashion group saw more non-figurative attributes in both shows compared to the non-fashion group.

Our general conclusion is that to see how people who are familiar with fashion understand photos from fashion shows, simply analyzing the straightforward attributes (non-figurative interpretations) of the depiction of humans in fashion images is not enough. The distinctive feature of the fashion group in understanding a fashion show is reflected in the metaphorical and metonymic interpretations. The core of figurative interpretation is how people derive new meanings from a form (Indurkhya, 2007). It is a way to have rich meaning with a limited number of forms.

More specifically, we argue that the two shows differ significantly in the following ways. First, with respect to figurative interpretations, the fashion group had more metonymic interpretations for the letter collection. We suggest that this is because the fashion group focused on the shape of the letter and the texture of the garbage. The difference between the shape and the texture is reflected in the language system as the difference between countable and uncountable nouns or the difference between nouns in English and Japanese (Nomura, 1996). Moreover, some studies have shown that young children who just acquired a language are sensitive to the differences between shape and texture (Soja et al., 1991), and based on the acquired language, there is a difference in the importance of features (Imai and Gentner, 1997). It is possible that people who are familiar with fashion are more sensitive to shape.

Second, in the non-figurative interpretations, the participants characterized the garbage show photos with outer traits and the letter show photos with inner traits. We suggest that this difference emanates from the concrete versus abstract contrast between the two shows rather than the texture versus shape contrast.

Needless to say, this research has some limitations. For example, by using four pictures from each show, we were able to capture only some parts of the information related to fashion design in a fashion show. Also, since we only targeted one fashion school, where the focus is on creative aspects of fashion, we were unable to ascertain what would have happened had we included participants from other fashion schools that teach more practical or commercial aspects of fashion. These points should be taken into consideration in the following studies.

For future research, we plan to study two points: (1) how the shape versus texture and the abstract versus concrete contrasts work; (2) whether the different responses to the shows by the fashion group with figurative interpretations were related to having more non-figurative interpretations.

Finally, we would like to make two comments based on our results from a more global perspective. One is from a human-agent interaction perspective and the other is a perspective from communication studies.

How people interpret a rich human image from the photo of a human, i.e., without interacting with the human, seems to share the same mechanism as how people see a non-human as a human (personification). Many researchers have explored why we personify computers (Nass, 1996; Colburn and Shute, 2008; Nass and Yen, 2010), robots, and androids (Straub et al., 2010). Our results suggest that by manipulating clothes or appearances as well as by training the human who interacts with agents, we can make agents appear more humanlike.

From the perspective of human communication, the target of this analysis is interesting because while we saw fashion shows as a form of communication between the designer and the audience in conveying human images, the purpose of this communication was not to convey the image in a precise manner. The purpose was communication itself, which was done by sharing the human image. Even with natural languages, we do not always try to convey messages precisely. For example, when we are playing with a language (Crystal, 1998), we are simply communicating for the sake of communicating. This type of activity can often lead to the creation of new words (Schmid, 2008, 2016). However, most linguistic analyses focus on communication as an exchange of information. Even in the framework of experimental semiotics (Galantucci, 2005; Steels, 2006; Kirby et al., 2008; Scott-Phillips and Kirby, 2010; Galantucci and Garrod, 2012), where the aim is to understand the emergence of communication or language by observing the usage of artificial communication tools, the main focus is on communication or a language that delivers information (Uno et al., 2012). As Clark (1997) points out, humans can extend their cognitive capacity using artifacts such as mobile phones, search engines, or language. Fashion is also an artifact for us to extend ourselves. By analyzing metaphor and metonymy in fashion, we can arrive at clues to analyze fashion as a phenomenon that reflects our communication capacity. We can expand our study of fashion design to reveal the mechanisms underlying our communicative abilities in general.

## Author Contributions

RU, EM, and BI conceived the experiments. RU performed the experiments. EM carried out the data analyses. All authors discussed and interpreted the results and contributed to drafts of this manuscript.

## Conflict of Interest Statement

The authors declare that the research was conducted in the absence of any commercial or financial relationships that could be construed as a potential conflict of interest.
