# Supplementary material for: Analyzing Visual Metaphor and Metonymy to Understand Creativity in Fashion
Source: Front Psychol. 2019 Jan 7;9:2527. doi: 10.3389/fpsyg.2018.02527 (PMC6330283; doi:10.3389/fpsyg.2018.02527)
Supplement: Supplementary file 1 [file Table_1.docx]

Supplementary Material

Analyzing Visual Metaphor and Metonymy to Understand Creativity in Fashion

Ryoko Uno^*^, Eiko Matsuda, Bipin Indurkhya

*** Correspondence:** Ryoko Uno

ryokouno@cc.tuat.ac.jp

The ratings by the three coders (A, B, C) are shown in the file “Table 2”. The coders rated the answers collected from the two groups of participants (fashion group and non-fashion group) using the inquiry sheets for the photos from the fashion shows (garbage collection and letter collection). The question on the inquiry sheet was as follows: “The following four pictures are from a fashion show. Look at the third large photo and answer what kind of *human* is represented by this photo.”

The answers by the participants were divided into “expression units” when they included more than one statements in their answers. For instance, in example (1), the answer includes two separate statements and was, thus, divided into (1a) and (1b). They were counted as separate expression units and were rated separately. The figures in the leftmost cells in the Table 2 file show the number of expression units.

(1)

a. *seibetsu o kanji-sase-nai*

gender ACC feel-CAUS-NEG

“It is a gender-neutral person.”

b. *ereganto de yasashii nihon-koten o kanjiru*

elegant and gentle Japanese-classic ACC feel

“I feel the person having an elegant and gentle Japanese classic style.”

In rating the expression units, those that were judged by the coders to be unratable because they did not describe a human image (such as “I don’t know” or comments about the designer) were marked with “X”s in the “ratability” row and were excluded from the subsequent rating process. For the remaining expression units, the coders were asked to rate how well each answer (the description of human image) matched the attributes in the six categories: metonymic, metaphorical, physical, personal, relational, and social role. Each rating was carried out using a six-point Likert scale (1 = *matches completely*, 6 = *does not match at all*).
